# Supplementary material for: in situ observation of reversible phase transitions in Gd-doped ceria driven by electron beam irradiation
Source: Nat Commun. 2024 Sep 17;15:8156. doi: 10.1038/s41467-024-52386-3 (PMC11408598; doi:10.1038/s41467-024-52386-3)
Supplement: Supplementary file 1 — Supplementary Information [file 41467_2024_52386_MOESM1_ESM.pdf]

Supplementary Information for

in situ observation of reversible phase transitions in Gd-doped ceria driven by electron beam irradiation

Ke Ran,[1,2,3*] Fanlin Zeng,[4] Lei Jin,[2] Stefan Baumann,[4] Wilhelm A. Meulenberg,[4,5] Joachim Mayer[1,2]

[1] Central Facility for Electron Microscopy GFE, RWTH Aachen University, Aachen 52074, Germany

[2] Ernst Ruska-Centre for Microscopy and Spectroscopy with Electrons ER-C, Forschungszentrum Jülich GmbH, Jülich 52428, Germany

[3] Advanced Microelectronic Center Aachen, AMO GmbH, Aachen 52074, Germany

[4] Institute of Energy and Climate Research IEK-1, Forschungszentrum Jülich GmbH, Jülich 52428, Germany

[5] Faculty of Science and Technology, Inorganic Membranes, University of Twente, Enschede, AE 7500, The Netherlands

* Correspondence: ran@gfe.rwth-aachen.de

Supplementary Note 1: Calibration of the effective Gd substitution of Ce

For the $Ce_{1-x}Gd_xO_{2-\delta}$ in our study, nominally 20% of the Ce cites are occupied by Gd (CGO20). As reported in our previous studies, additional phase was formed during the sintering, which consumed considerable Gd and resulted in a much lower effective Gd substitution.[1] Taking the CGO10 as a reference, where no additional phase is detected and thus the effective Gd substitution can be considered as 10%, the actual Gd composition in the nominal CGO20 is estimated as ~12%, as in Fig. S1.

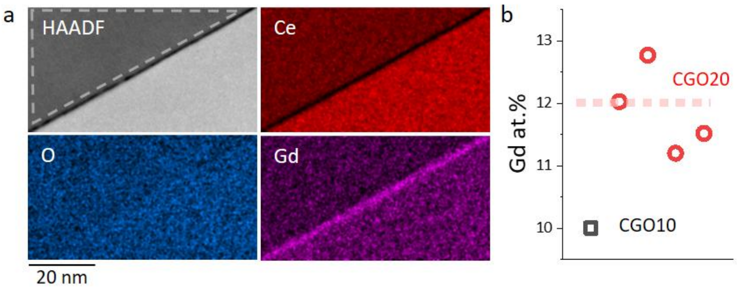

Supplementary Figure 1: (a) The energy-dispersive X-ray spectroscopy (EDXS) chemical mapping results around a CGO10 interface: HAADF image and the elemental maps in intensity from the Ce *L* line, Gd *L* line, and O *K* line. Only slight Gd segregation thinner than 2 nm is noticed along the interface. The region defined by the dashed triangle in the HAADF image is used as a reference. Similarly, EDXS chemical mapping were recorded from several CGO20 grains, and the calibrated Gd compositions are listed in (b). A mean value of ~12% is estimated.

Supplementary Note 2: Probing the F-type structure by STEM

In our experiment, the phase transition was only observed under the TEM imaging condition, but not STEM. As discussed in the text, the observed phase transition can be considered as a collective rearrangement of a large number of $V_O^{\cdot\cdot}$ paired with Ce reduction. The ultrafine probe (< several Å) in STEM mode is thus unlikely to activate enough $V_O^{\cdot\cdot}$ at the same time, and cause detectable structural changes in our case. For CeO_2 nanoparticles, STEM induced phase transition has been demonstrated, but a critical dose rate must be exceeded.[2] The mechanism behind is that the $V_O^{\cdot\cdot}$ created by exposure to electron beam are actively annihilated as the sample re-oxidizes in the microscope environment. As a result, only when the rate of vacancy creation exceeds the recovery rate will beam damage begin to accumulate. Obviously, such a dose rate threshold is not exceeded in our case, as only F-type structure is determined here. The images in Fig. S2 were recorded from the same CGO grain at different high voltages and with different imaging conditions. Phase transition can be easily induced under the TEM condition either with 200 kV or 300 kV accelerating voltage (also 60 kV as in Fig. S7), evidenced by the extra spots in the FFT patterns as marked by the rings. In contrast, the simultaneously acquired HAADF and iDPC images suggest pure F-type structure.

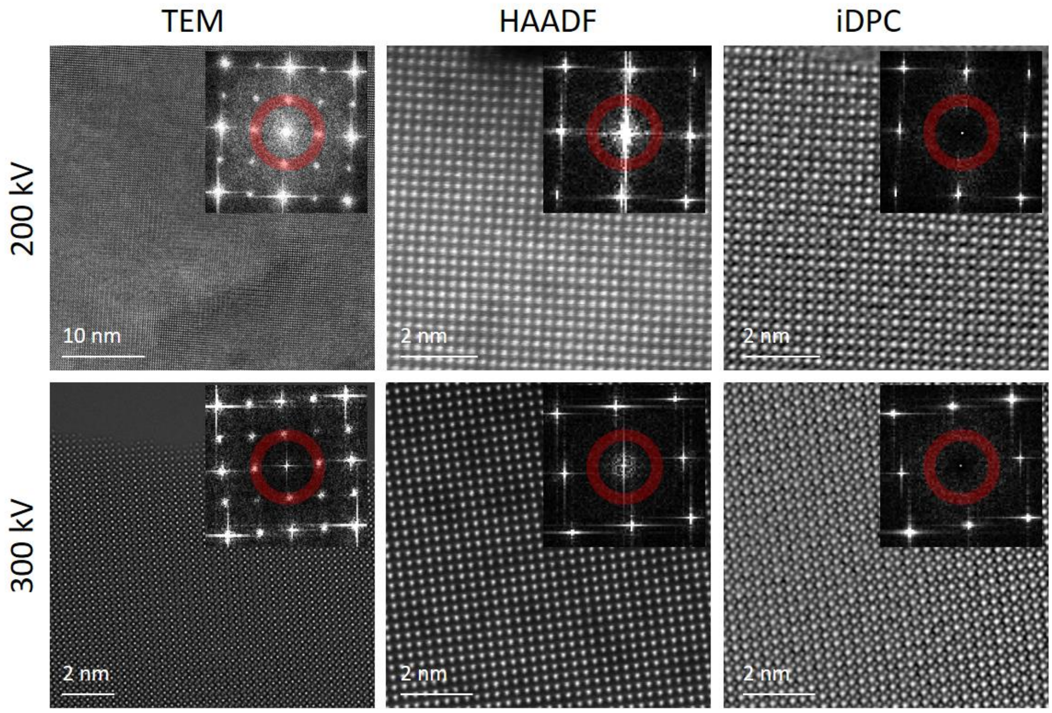

Supplementary Figure 2: The same CGO grain imaged with different accelerating voltages and illumination conditions along <001>. The upper-right insets are the corresponding FFT patterns from each image.

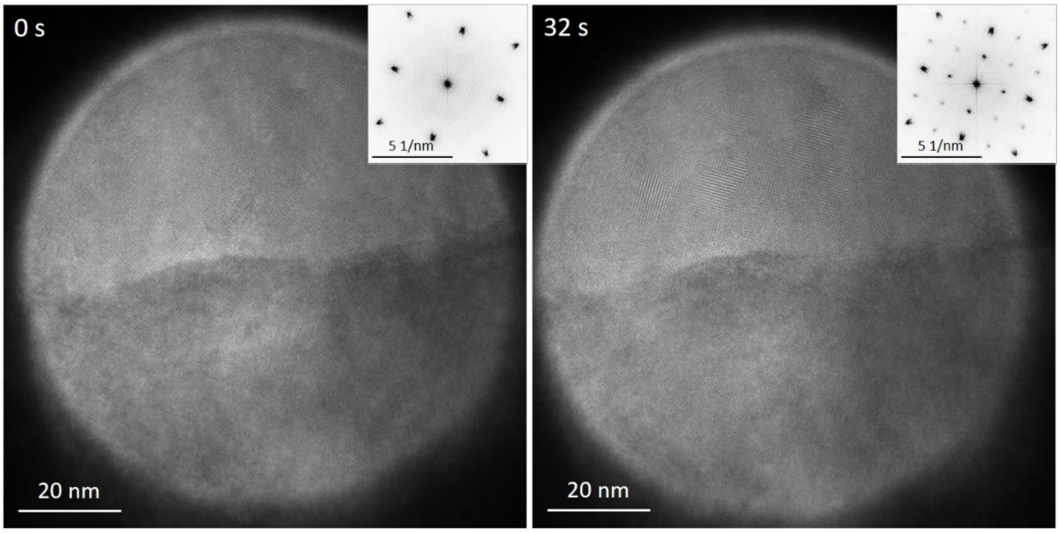

Supplementary Figure 3: The recorded HRTEM images at different time corresponding to Fig. 3a-b. Each image has a size of 4k*4k with ~0.025 nm/pixel.

Supplementary Note 3: Orientation of C-type by FFT

Experimentally, based on the FFT, the split of r_A and r_B is constantly detected at a later stage of all the F-to-C transitions, as in Fig. 3f. Taking the structure of $Ce_{0.125}Gd_{0.875}O_{1.562}$[3] as an example, HRTEM image is simulated along [001] in Fig. S4a. The corresponding FFT pattern in shown in Fig. S4b. Two directions, A and B, are defined in Fig. S4b, and Fig. S4c plots the intensities along each direction. Following the F-type structure, the {010} spots along A are evidently stronger than those along B, while the {020} spots are more or less comparable between the two directions. Besides, diffraction pattern based on dynamical theory is simulated in Fig. S4d as well. Consistent with the FFT pattern, the (010)/(0-10) spots are much stronger than the (100)/(-100) spots. According to the simulations, the stronger {010} spots in either FFT or diffraction pattern are associated with the direction in real space, along which both the M layer intensity and d_O oscillate. Therefore, the {010} spots with different intensities in FFT, and consequently the split of r_A and r_B measured from experiment, are able to shed light on the orientation of the C-type.

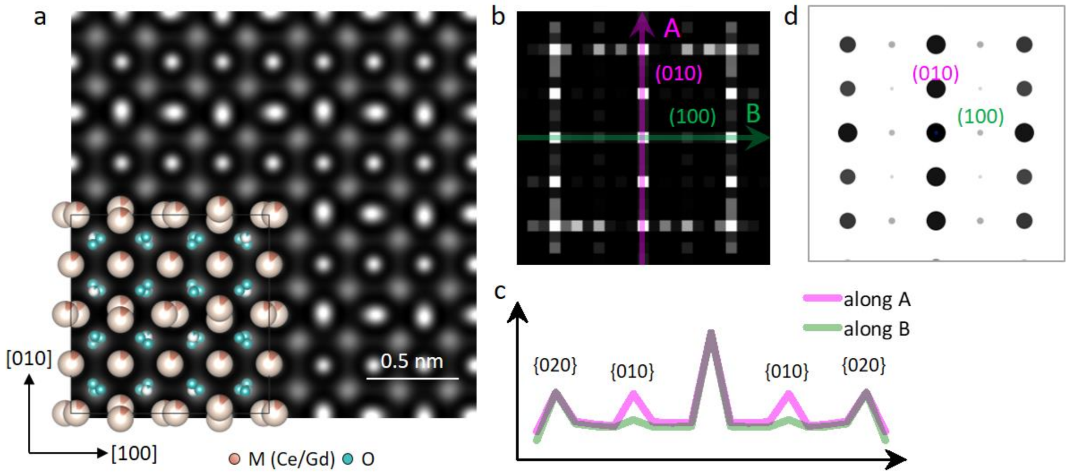

Supplementary Figure 4: (a-b) The simulated HRTEM image and its corresponding FFT pattern based on $Ce_{0.125}Gd_{0.875}O_{1.562}$ along [001]. A structural model is overlaid on the image. (c) The line profiles along the A and B as outlined in (b). (d) The simulated diffraction pattern from $Ce_{0.125}Gd_{0.875}O_{1.562}$ based on dynamical calculation. All the indexing is following the F-type symmetry, and a sample thickness ~10 nm is used.

Supplementary Note 4: HRTEM simulation on C-type models

Based on the existing C-type models, $Ce_{1-x}Gd_xO_{2-\delta}$ $(0.313 \leq x \leq 1, \delta = x/2)$,[3-5] HRTEM images are simulated under our experimental condition. d_{M_v} and d_{O_v} are then estimated from the simulations. Fig. S5a-b show two examples with $\delta = 0.219$ and $\delta = 0.375$. Fig. S5c plots the determined d_{M_v} and d_{O_v} by averaging within each labeled layer. Similar to Figure 4b, d_{M_v} is almost constant for all the layers, while d_{O_v} oscillates regularly. The amplitude of d_{O_v} oscillation is also found much larger for $\delta = 0.375$ than for $\delta = 0.219$.

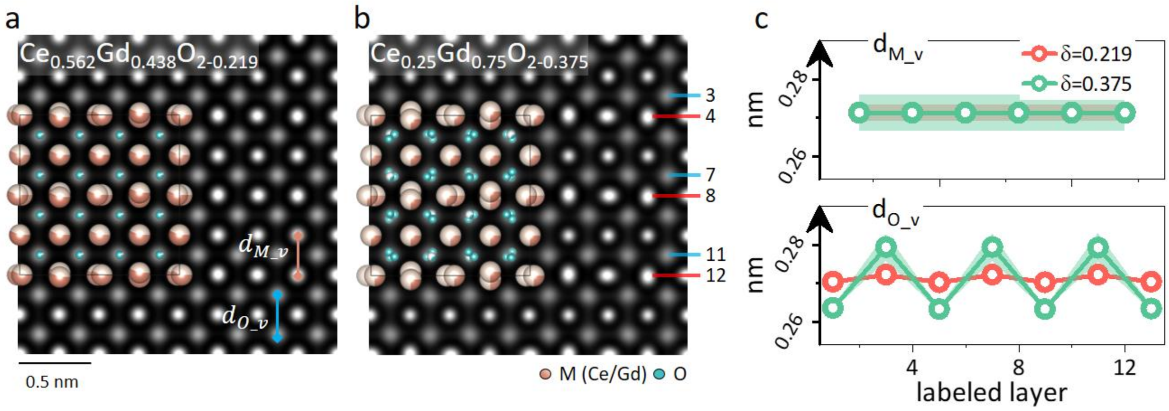

Supplementary Figure 5: (a-b) Simulated HRTEM images based on two C-type models with different compositions. (c) The estimated d_{M_v} and d_{O_v} from a-b.

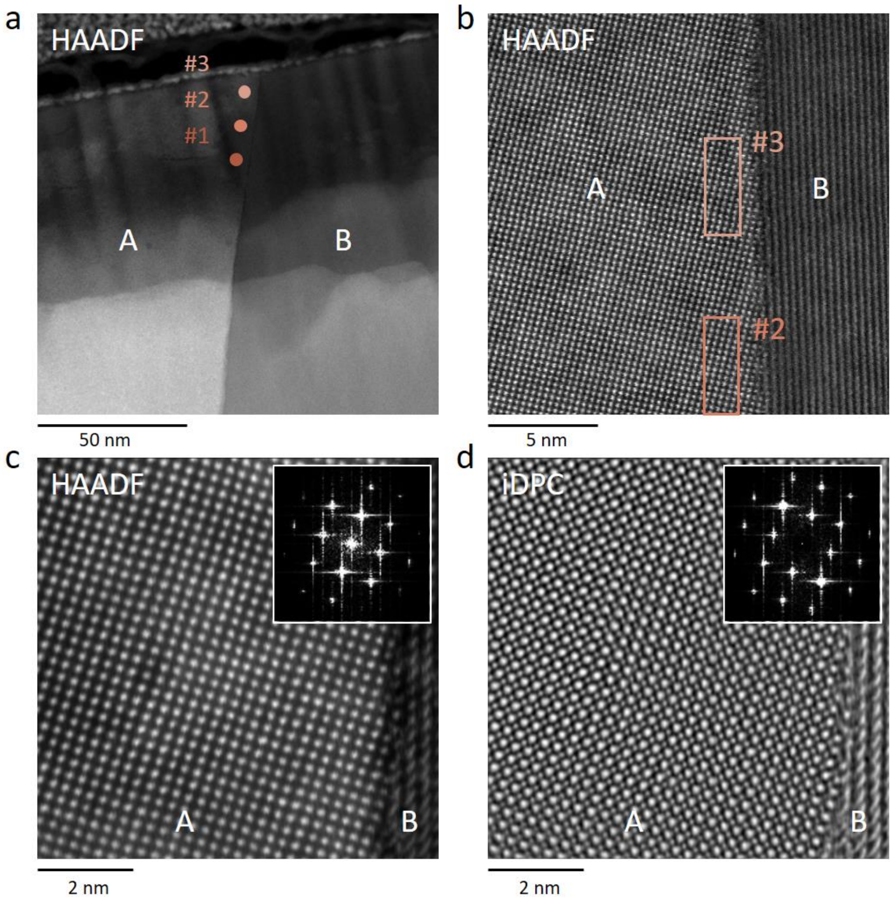

Supplementary Figure 6: (a) HAADF image shows two CGO grains (A and B). Three locations are marked, corresponding to the three EELS curves in Fig. 6c. (b) HAADF image recorded between collecting EELS signal from location #2 and #3. The scanning regions for #2 and #3 are outlined by the rectangles. (c-d) The simultaneously acquired HAADF and iDPC images recorded around the three locations after the EELS measurement. No extra spots can be noticed in the FFT patterns (upper right insets), suggesting no significant structural changes caused by STEM imaging or EELS measurements.

Supplementary Note 5: Estimation of the temperature raise under electron beam

The local temperature rise is resulting from energetic dissipation of the electron beam (e-beam) travelling through the thickness of the CGO.[6] The generated heat is transferred to outside the irradiated region by conduction through the cylindrical surface area $S = 2\pi rt$, where r is the radius of the e-beam, and t is the thickness of the irradiated CGO. The heat then travels through the CGO over a distance R until it reaches the Cu bar, where the temperature is assumed to be at ambient ($T_0 = 298\ K$). For our lamella, $R \approx 6\ \mu m$, and r varies.

At steady state, the heat generation rate is balanced by the heat transfer rate, and the highest temperature, T_{max}, is at the center of the beam by

$$T_{max} = T_0 + \frac{j\rho}{2e\lambda}\frac{\Delta Q}{\Delta x}r^2 \ln\frac{R}{r} \quad (1)$$

where e is the electronic charge of $1.6 \times 10^{-19}\ C$, ρ is the density of ceria $7.215\ \ g/cm^3$, and λ is the thermal conductivity of ceria $14.2 \times 10^{-2}\ J \cdot K^{-1} \cdot cm^{-1} \cdot s^{-1}$.

$\frac{\Delta Q}{\Delta x}$ is the energy dissipated per unit mass thickness, and can be estimated by

$$\frac{\Delta Q}{\Delta x} = 7.8 \times 10^4 \cdot \frac{Z}{A} \cdot \frac{1}{E} \cdot \ln\frac{E}{J} \quad (2)$$

where Z and A are the atomic number and atomic mass number for ceria. E is the electron energy of $2 \times 10^5\ eV$, and $J \approx 13.5Z$ is the mean ionization energy.

The effective atomic number (Z_{eff}) and effective atomic weight (A_{eff}) of a heterogeneous material is[7]

$$Z_{eff} = \sum_i (p_i \cdot Z_i / A_i) / \sum_i (p_i / A_i) \quad (3)$$

$$A_{eff} = 1 / \sum_i (p_i / A_i) \quad (4)$$

where Z_i and A_i are the atomic number and atomic weight of the ith element, and p_i is the fractional part by weight of the whole mixture occupied by the element with atomic number Z_i.

For ceria, $Z_{eff} = 24.67$ and $A_{eff} = 57$ are estimated, leading to[8] $\Delta Q/\Delta x = 1.0799\, eV \cdot cm^2 \mu g^{-1}$. Taking the EDR readouts from the microscope and measuring the beam radii from the TEM images, the temperature rises for the transitions shown in Figure 2e are listed in the the Supplementary Table 1.

	Beam radius (nm)	EDR readout ($e \cdot Å^{-2} \cdot s^{-1}$)	Normalized EDR	$\Delta T = T_{max} - T_0$ (K)
T1	42	3825	1	0.014
T2	48	2656	0.69	0.013
T3	65	1700	0.44	0.014
T4	94	1153	0.3	0.019

Supplementary Table 1: the estimated temperature change for each F-to-C transition.

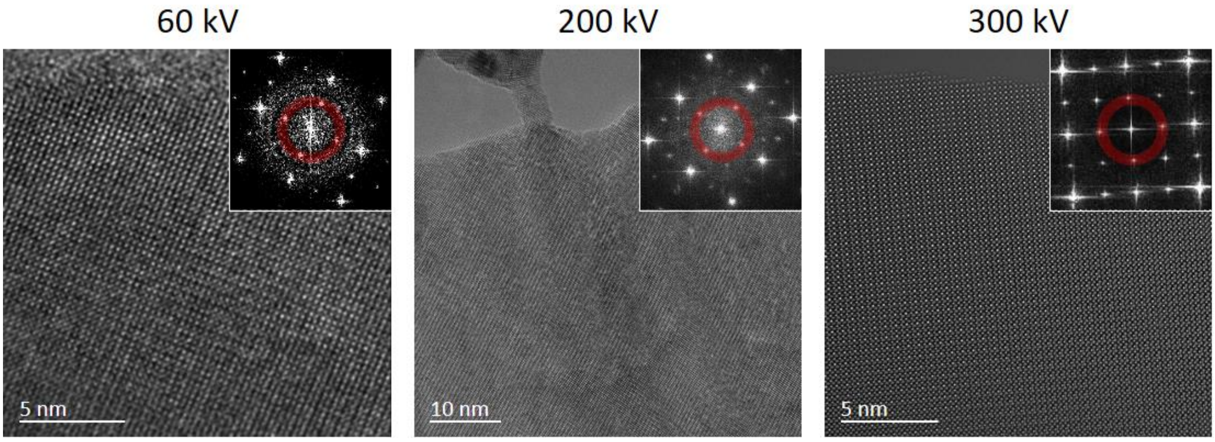

Supplementary Figure 7: Comparison between the TEM images recorded with different accelerating voltages. Insets are the corresponding FFT patterns. As marked by the rings, extra spots show up for all the images, suggesting successful F-to-C transitions with all voltages.

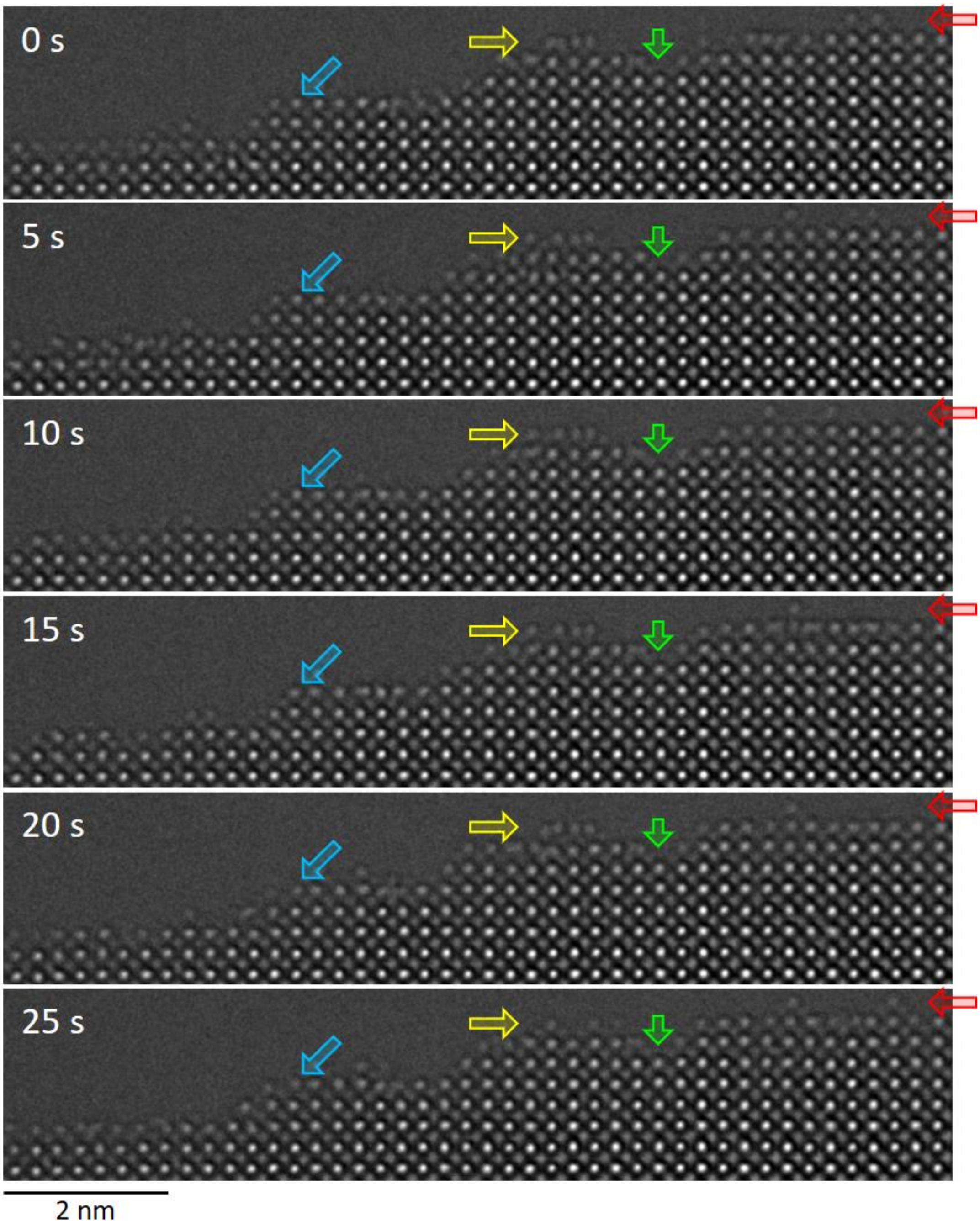

Supplementary Figure 8: A series of HRTEM images with 300 kV tracking possible beam damage. Four sets of arrows are overlaid on the image, and each is marking the same position in the images. At the surface layers, atomic rearrangements are noticed, as suggested by the red, yellow, and blue arrows. Any significant changes especially within the bulk region, the green arrows, is not detected.

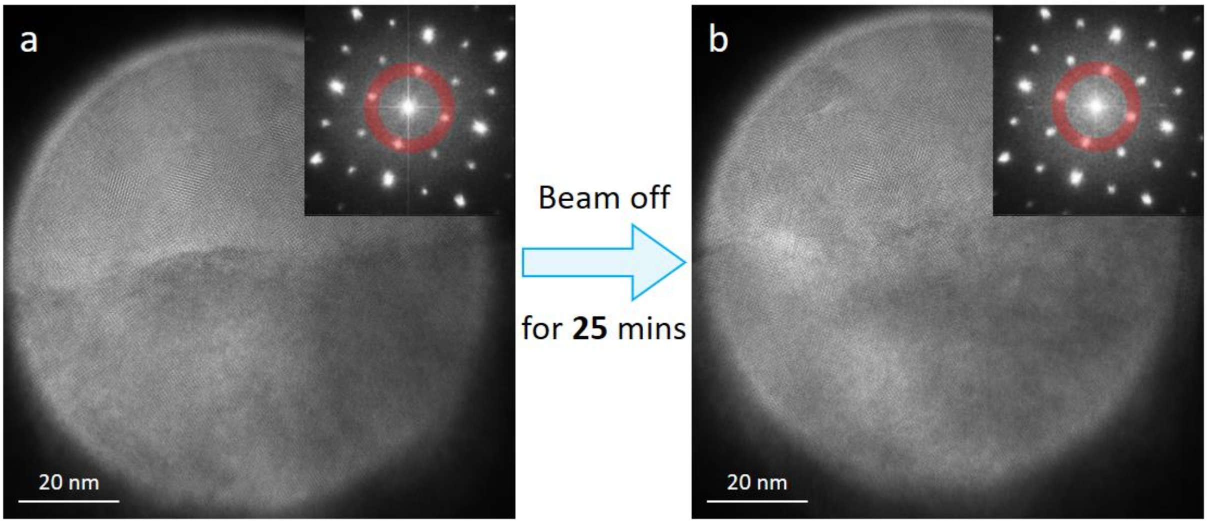

Supplementary Figure 9: TEM images of the C-type structure before and after blanking the beam for 25 mins. (a) The CGO is adopting the C-type structure as strong {010} spots showing up in the FFT pattern in the upper-right inset. (b) The same region after blanking the beam for 25 mins. Comparable {010} spots are detected in the FFT pattern again, suggesting that the C-type structure is rather stable without external stimulus. Slight sample drift is noticed between (a) and (b), as (b) was immediately acquired once the beam is un-blanked, to avoid any driving force for a F-to-C transition. The exposure time for both images is 0.04 s.

Supplementary References

1 Ran, K. *et al.* The *in situ* generated emerging phase inside dual phase oxygen transport membranes. *Acta Mater* **234** (2022).

2 Johnston-Peck, A. C., DuChene, J. S., Roberts, A. D., Wei, W. D. & Herzing, A. A. Dose-rate-dependent damage of cerium dioxide in the scanning transmission electron microscope. *Ultramicroscopy* **170**, 1-9 (2016).

3 Scavini, M., Coduri, M., Allieta, M., Brune, M. & Ferrero, C. Probing Complex Disorder in $Ce_{1-x}Gd_xO_{2-x/2}$ Using the Pair Distribution Function Analysis. *Chem Mater* **24**, 1338-1345 (2012).

4 Scavini, M. *et al.* Percolating hierarchical defect structures drive phase transformation in $Ce_{1-x}Gd_xO_{2-x/2}$: a total scattering study. *Iucrj* **2**, 511-522 (2015).

5 Grover, V., Achary, S. N. & Tyagi, A. K. Structural analysis of excess-anion C-type rare earth oxide:: a case study with Gd1-xCexO1.5+x/2 (x= 0.20 and 0.40). *J Appl Crystallogr* **36**, 1082-1084 (2003).

6 Reimer, L. & Kohl, H. *Transmission Electron Microscopy: Physics of Image Formation*. (Springer, 2008).

7 Murty, R. C. Effective Atomic Numbers of Heterogeneous Materials. *Nature* **207**, 398-& (1965).

8 Heo, A. E. Y., Torres, D. D., Banerjee, P. & Jain, P. K. In-situ electron microscopy mapping of an order-disorder transition in a superionic conductor. *Nat Commun* **10** (2019).
